# The Adaptive Proculturation Process of Being a Psychotherapist as a Kazakh Asylum Seeker in Sweden

**DOI:** 10.1007/s42087-022-00294-7

**Published:** 2022-06-01

**Authors:** Björn Boman

**Affiliations:** Department of Education, Stockholm University, Frescativägen 54, 106 91, Stockholm, Sweden

**Keywords:** Proculturation, Psychotherapists, Asylum seeker

## Abstract

The term acculturation is important for describing and analyzing how for example migrants gradually become accustomed to a new host society. The term proculturation is similar but emphasizes the real-life experiences of migrants, as well as the fusion between familiar and unfamiliar ideas, things, and experiences. However, so far there is a dearth of studies which have aimed to explore such a construct empirically. The current article used a limited but meaningful example, the lived experiences of a Kazakh migrant in Sweden whose occupation is being a psychotherapist. The aim was to understand the cultural identity of this individual as regards processes of migration from A (Kazakhstan) to B (Sweden), as well as related proculturation processes. Moreover, the focus was also on the specific and precarious work conditions for a person who does not have the possibility to work officially as a psychotherapist during an extensive residence permit application process. Information derived from a semi-structured interview indicates that basic cultural identity markers (woman, Kazakh, Russian-speaking, Muslim) remained constant after residing in Sweden for more than 5 years but that some elements of the more secular-liberal Swedish culture (e.g., the Swedish language, increased alcohol consumption) were appropriated. The person used social media apps like WhatsApp as a technological tool to practice the profession as a psychotherapist in a transnational setting, which constitutes a different strategy than how Swedish as well as Kazakh psychotherapists generally perform this profession.

## Introduction

The term proculturation was introduced by Gamsakhurdia (2018) to signify a more dynamic and nuanced understanding of cultural identity than the term acculturation could supposedly offer. Acculturation does generally mean the gradual acceptance (or in some cases rejection) and absorption of cultural and social elements in new countries/locations and is typically measured by attitude-related items in quantitative research (e.g., Chirkov, 2009; see also Berry, 1997), whereas proculturation implies a fuzzier yet perhaps more insightful understanding and interrelationship between cultures A (i.e., the sending country and country of origin of migrant X) and B (i.e., the receiving country and new place of sojourn of migrant X) (Gamsakhurdia, 2018). While earlier contributions on proculturation by Gamsakhurdia (e.g., Gamsakhurdia, 2018, 2019, 2020) include some empirical examples and reflections, there is nevertheless a dearth of empirical studies which hinge on this more recent theoretical concept.

The current article aims to contribute with a limited amount of empirical data in this regard by using “small data” from a single Kazakh migrant in Sweden, who has worked unofficially as a psychotherapist in Sweden while waiting for a definitive decision during an extensive residence permit application process (2015–2022). Needless to say, the aim is not to convey something general about Kazakh migrants who reside in Sweden, nor even more specific sub-groups such as Kazakh migrants with a psychology-related profession. However, it is to show one way in which proculturation may take place. Moreover, it is to highlight how migrants may use flexible cultural, technological, and economic tools such as social media to function in a new society as regards job opportunities and specific professions. The current article has a rather broad take on culture (e.g., see Jahoda, 2012) and includes both the macro cultural variables (i.e., economic and political system, religious participation and demographics, see Ratner, 2017), intercultural encounters, and micro cultural semiotic processes (e.g., Valsiner, 2014) and how they interact with the macro cultural factors. Moreover, even material or technological tools may be used as cultural artefacts (e.g., Valsiner, 2014; Vygotsky, 1971). However, the specific outline is first and foremost related to proculturation processes (Gamsakhurdia, 2018, 2019, 2020).

The article proceeds with a background section, a methodology section, and an integrated analysis fleshed out in four sub-sections and ends with a conclusive discussion.

## Theoretical Background

### Conceptual Framework

As Valsiner (2014) notes, “Culture is not an entity, a “thing” that one “has,” or “gets” (by assimilation or socialization), but the active process of mediating human lives through signs, both intra- and inter-psychologically. The central issue for cultural psychology is to locate culture in the life activities of agentive persons. These persons are meaning-makers, and the meanings made frame their relations with the environment.”

The term acculturation is important for describing and analyzing how, for example, migrants gradually become accustomed to a new host society. The term proculturation, as suggested by Gamsakhurdia (2018, 2019), is similar but emphasizes the real-life experiences of migrants, as well as the fusion between familiar and unfamiliar ideas, things, and experiences (Gamsakhurdia, 2019). As such it does partly resemble cultural hybridization (e.g., Boman, 2021b; Pieterse, 2015) but focuses specifically on individuals’ adaptation processes within new environments. Another theoretical benefit with the concept of proculturation, compared to acculturation, is the multidirectional possibilities embedded in the concept (i.e., there might be more than two cultures that meet or influence an individual), as well as the possibility of semiotic transformation into a new synthesis related to the person’s identity and meaning-making processes. In other words, proculturation may “produce” or enable cultural novelties (Gamsakhurdia, 2019, 2020).

When aiming to come closer to the real, lived experiences of individuals, it is important to pay attention to nuances and what it may mean to move from place A to B. Gamsakhurdia (2019, p. 165) suggests:

So, emigration is the field where semiotic mediation might be intensified due to the change of a living environment; however, it does not necessarily imply mindblowing jump from one radically different cultural planet to another. Due to the intense globalization and spread of online mass media, information and technologies are shared so easily and so widely, that it is highly unlikely that any emigrant can find any host society/culture, which would be totally new/unfamiliar to him/ her. Most emigrants are already acquainted with potential host societies, at least up to some level.

In other words, a (young) person who is living in a country like Kazakhstan, especially larger cities like Almaty or the capital Nur-sultan (previously named Akmola, followed by Astana), tends to be familiar with global communication tools like Smartphones and social media apps, and through these and other sources, he or she might be aware of the general economic, political, social, and cultural conditions of the place that he or she seeks to move to at a later time. Hence, the interdependence between the old and the new elements of two or more cultures is crucial as regards proculturation theory (Gamsakhurdia, 2018).

### Literature Review

This earlier research section relates to autobiographical acculturation studies, the practice of psychotherapy in Kazakhstan and Sweden, and migration and/or national identity studies relevant for the Kazakh and Swedish contexts.

Using an autobiographical approach, Kwak (2010; see also Tartarovsky, 2010) described personal experiences in relation to acculturation research of East Asian migrants and stresses the mix of responsibility and freedom that signifies a liberal and multicultural city like Toronto, compared to the thoroughly monocultural home country South Korea. The aloneness position in the new location implied that this person had to construct a partly new “Self-I” as compared to the prototypical “We-I” in South Korea. While South Korea and Kazakhstan have many differences (e.g., religious, linguistic, ethnic), there are some overlaps in regard to traditional values like modesty (e.g., Boman, 2020; Danabayev & Park, 2020; Shim, 2001), as well as with regard to the transition from Korea/Kazakhstan to a western-liberal location described by Kwak (2010) and the interviewee in the current study (Toronto/Stockholm).

Manhica et al. (2018) have examined the labor market integration of young accompanied and unaccompanied migrants, often originating from Afghanistan, Syria, Somalia, Iran, and Iraq, in contemporary Sweden. They found that both young accompanied and unaccompanied migrants had difficulties to obtain a secure place in the labor market compared to native Swedes, even when they had acquired comparable educational levels as the majority group. Partly similar findings were discerned by Vogiazides and Mondani (2019), who found that fractions of migrants who resided at the extremes of a geographical spectrum (the capital Stockholm and smaller towns or villages) had it easier to find their first job compared to those with intermediate geographical positions. Boman (2021a) did also find negative associations between Swedish municipalities with large shares of non-natives and school results, in part likely because such categories of students also have socioeconomic and language difficulties.

Spehr and Kassenova (2012) analyzed factors which were related to having a stronger sense of loyalty to the current post-Soviet nationhood model of Kazakhstan. They found that being Asian and/or Sunni Muslim and having relative economic affluence were linked to having a stronger sense of loyalty to the recent construct of a Kazakh state and nationhood.

The current article adds to three strands of literature within interdisciplinary psychology, first and foremost cultural psychology, but also qualitative acculturation/proculturation studies, migration studies relevant for the Kazakh and Swedish contexts, as well as psychotherapy as a practice within different cultural and national contexts. As Smedslund (2009) observes, researchers may be informed by psychotherapy practitioners as regards for example sub-disciplines within psychology. Hence, the current contribution adds some understanding to the research–practice nexus within psychotherapy.

### Kazakhstan’s Economic and Cultural Profile

Kazakhstan is the ninth largest country in terms of size of its geographical area but has only approximately 19 million inhabitants. The country is located in Central Asia, neighboring, for example, Russia, China, and Uzbekistan. Kazakhs make up the largest majority group (68%), while people of Russian/Slavic descent constitute the largest minority group (19.3%) (World Factbook, 2022). During the period as a Soviet state, the country was inhabited by Crimean Tatars, Germans, and Koreans who had migrated there (Spehr & Kassenova, 2012). However, these days such groups constitute only small fractions of the total population (World Factbook, 2022).

In relation to politics and religion, Kazakhstan has transitioned into a democratic and secular country but has palpable autocratic features. About 70% do at least nominally identify themselves as Sunni Muslims and about 26% as Russian Orthodox Christians. This situation is vastly different compared to the atheist Soviet period (Edelbay, 2012; Spehr & Kassenova, 2012). Particular forms of Shamanism (Tengri, i.e., sky cult) or syncretic forms of religion (e.g., Islamic Sufism/Shamanism) are also part of the sociohistorical and religious landscape (Edelbay, 2012). However, typically, people of a Kazakh identity are rather a-religious (Edelbay, 2012; Spehr & Kassenova, 2012).

The annual growth rates have been substantial after the dissolution of the Soviet Union in 1991, and currently Kazakhstan has a GDP per capita which is higher than former Yugoslavian countries such as Serbia, Bosnia, Montenegro, and North Macedonia and slightly higher than Turkey (World Bank, 2020). The economic development is largely related to natural resources such as oil and gas, as well as foreign investment (World Factbook, 2022).

Culture-wise, one may also notice a co-existence of the Kazakh language and Russian language, as well as a gradual transition towards English as a third important language in regard to general education, international relations, and work contexts. A gradual transition towards de-Russification of the Kazakh language, signified by a switch from the Cyrillic to the Latin alphabet no later than 2025, is planned by the state’s government. This process is also associated with modernization, globalization, and westernization (Yergaliyeva, 2018). Moreover, the youth aspects of Kazakh/Qazakh culture have recently been influenced by Korean popular culture such as K-pop. The local and Kazakh version of K-pop music, referred to as Q-pop, signifies a hybrid of Kazakh, Korean, and Western culture (Danabayev & Park, 2020; see also Boman, 2021b, 2021c).

### Sweden’s Economic and Cultural Profile

Sweden is located in Scandinavia, in northern Europe, and has approximately 10 million inhabitants. It is a wealthy and democratic welfare state, which is characterized by a hybrid of social democratic welfare policies and neoliberal capitalism. The country is export-oriented and has a strong base in large industrial companies (World Factbook, 2022; Sanandaji, 2020; see also Bunar, 2010). The culture is described as one if not the most individual-secular state in the world (World Values Survey, 2022). Historically, it does also have strong egalitarian features (Sanandaji, 2020). However, the multicultural situation has led to more complex conditions in terms of religious pluralism and cultural values as secular individualism co-exists with, for example, Islam these days (Boman, 2021b).

### Migration Policies in Sweden

Since the introduction of a multicultural model in 1975 (Regeringen, 1975), Sweden has had a liberal migration policy, typically supported by both social democratic, liberal, and liberal-conservative parties. For instance, Sweden has accepted both work migrants and refugees from, for instance, Greece, Turkey, Chile, Iran, and Yugoslavia, of which the largest shares of these national groups have been well integrated in regard to education and earnings (Boman, 2021b).

However, the 1990s onwards there has been a large influx of low-skilled migrants to Sweden (Ekberg, 1999). Moreover, economists such as Sanandaji (2020) and migration scholars like Vogiazides and Mondani (2019) have argued that the socioeconomic integration of migrants in Sweden has typically been unsuccessful, especially in the three largest cities Malmö, Göteborg, and parts of Stockholm, which has led to an increased fiscal burden, high unemployment rates and crime rates (Sanandaji, 2020). In the aftermath of the migration crisis in 2015, which affected Sweden quite significantly, the migration policy in Sweden has become more restrictive, but Sweden remains less so compared to neighboring Denmark and Norway (Beck et al., 2017).

Migrants who are granted a residence permit in Sweden do typically come from Iraq, Syria, Afghanistan, Eritrea, and Somalia and less rarely do they originate from post-Soviet states in Central Asia, although a few hundred people come from, for example, Turkmenistan, Kyrgyzstan, Uzbekistan, and Kazakhstan. These were granted citizenships related to family association, work, studies, or asylum (Swedish Migration Agency, 2020). Thus, Kazakhstan is far from a typical sending country of migrants in relation to Sweden (and thus Sweden is not a standard receiving country in that respect), but it is possible to obtain a permanent residence permit and citizenship in Sweden for some.

### Differences Between the Psychotherapeutic Profession in Kazakhstan and Sweden

According to Nurmagambetova and Assimov (2016), there were 33 psychiatric clinical settings in Kazakhstan in 2015. In 2013, there were 14 psychiatric hospitals, and these included in total 5044 beds. The number of beds increases to nearly 10,000 if related categories of psychiatric treatment are included in the statistics. Around 2015, there were only 72 active psychotherapists in Kazakhstan. Overall, up until 2015, the mental healthcare field (i.e., both psychiatry and clinical psychotherapy) in Kazakhstan suffers from several structural and material deficiencies (Nurmagambetova & Assimov, 2016).

This might be compared to Sweden which currently has about 5700 registered psychotherapists (Samrådsforum, 2022). That may imply, for example, that either psychotherapy is an emerging profession in Kazakhstan which requires more practitioners to fill the demand for such specialists or that psychotherapists are pushed out of the country in order to seek more promising and well-paid work opportunities elsewhere.

With regard to the representation of various psychotherapy perspectives and practices, these are found in both countries (Larsson et al., 2009; Nurmagambetova & Assimov, 2016;), but because of the far greater number of practitioners in Sweden, both particular approaches such as cognitive behavioral therapy (CBT) and a psychodynamic approach, as well as integrated perspectives, are more common and widespread there (e.g., cf., Larsson et al., 2009; Nurmagambetova & Assimov, 2016; Socialstyrelsen, 2022; Samrådsforum, 2022).

## Methodology

The current article builds on a combination of a semi-structured interview (the author interviewed a psychotherapist from Kazakhstan who resided in Sweden as an asylum seeker) and semi-autobiographical narration (specifically, the author compiled a narrative based on the information retrieved from the interviews with the interviewee). In concordance with, for example, discourse analysis and recent examples of qualitative content analysis (e.g., Fairclough, 2003; Krippendorff, 2018), the sociocultural, sociohistorical, and economic contexts and earlier literature were used to situate the autobiographical narrative patterns in a meaningful way and corroborate or contextualize the findings from other sources. Gamsakhurdia (2018, 2019, 2020) does not suggest any specific limitations on how proculturation might be applied methodologically, but the author has followed a qualitative and hermeneutic approach in that regard which is akin to the earlier articles that have elaborated the concept and its application.

The semi-structured interview, which was used in this case (e.g., Bryman, 2011; McIntosh & Morse, 2015), aimed to collect basic information about age, educational trajectory, reasons for migrating, years of duration in Sweden, cultural self-identification, and how the work situation had been constituted during the years of residence in Sweden. For example, the author aimed to know more about how the interviewee could conduct the work while being an asylum seeker and to which extent the opportunity to perform the psychotherapy profession abroad was a main factor for the decision to migrate (to Stockholm, Sweden, in 2015). In addition, it included questions about culture and identity. For example, the author asked the interviewee what basic self-described national identity she predominantly had before as well as during the period of residence in Sweden and how Swedish culture had affected the primary identity (e.g., Kazakh).

In line with McIntosh and Morse (2015), the position taken by the author was mainly interpretive/descriptive, meaning that the author aimed to describe patterns with precision but also interpret proculturation processes based on the interviewee’s sharing of information. This implies that the theoretical context influenced the interpretation of the data (Bryman, 2011; Krippendorff, 2018). Specifically, the meaning-making and semiotic processes of the interviewee were highlighted in regard to the analysis and presentation of the interview data (Gamsakhurdia, 2020).

The interview was held in Swedish in the first half of 2021 and lasted for approximately 60–75 min, but follow-up interactions included more written and oral communication. The latter form of communication was related to clarification and participant checking of the interview data. The author and the interviewee had previously met in informal contexts, and as the author considered the experiences of the interviewee to be interesting and meaningful to share, it was important to bring together and formalize such fragmented notions into a more focused setting and narrative (i.e., an interview). The interviewee agreed to participate as regards this suggested research initiative.

A prerequisite for the presence of fact-checking of information presented in simple Swedish and the narratives compiled and written in English was that the author was transparent and clear about the included information and what it meant and even used some Russian concepts and back-and-forth translations (from English to Russian) to avoid misinterpretations and misrepresentations. Nonetheless, this procedure comes with some limitations in terms of discrepancy between the two different selves’ interpretive frames (e.g., see Valsiner, 2014; Gadamer, 2013). The author could never mirror the exact interpretation of the interviewee, and this individual’s specific life situation. On the other hand, the author could in part use a more unattached and “objective” approach which comes with some advantages (e.g., Bryman, 2011; Ratner, 2002).

### Research Ethics

The procedures that underlie the crafting this article, as well as its content, conform to the ethical guidelines stipulated by the Swedish research council (the Swedish Research Council, 2017). Key principles and criteria include consent (i.e., any participant must have the possibility to give consent in order for the research project to proceed), confidentiality (i.e., not disclosing any participants’ official identity), information (i.e., providing adequate information about the aim and purpose of the research and what it implies to participate), and utilization (i.e., the data collected through for example interviews or questionnaires will only be used for research purposes and often stored in accordance with specific data storage guidelines).

In addition, the author paid attention to transparency and participant checking (Tracy, 2010) as well as to not disclose too much information (about the interviewee) in order to consider the interviewee’s vulnerable position as a female migrant whose residence application had been rejected. That is also why quotes are somewhat sparse in the current article, as these might disclose information about the specific parlance of the interviewee. The author has also left out information about the interviewee’s specific ethnic background, which is of mixed origin, as well as number of siblings, specific hobbies, and part-time jobs, as they might disclose the identity of this person.

## Findings

### Proculturation Processes in a New Country and Culture

After 4 years of studies in clinical psychology in Almaty, Kazakhstan, inclusive of a psychotherapy practice period in Moscow, Russia, the interviewee, then in her early 20 s, decided to go to Sweden in 2015. This was a year which has become known as the “migration crisis” in Sweden (e.g., Beck et al., 2017). That decision was made in order to seek the opportunity to obtain a residence permit (“uppehållstillstånd” in Swedish) as an asylum seeker or as a work-related migrant if that could turn out to be an option. The decision was made to be able to permanently move to a country with better opportunities than Kazakhstan could offer in terms of quality of life and work-related prospects. However, the practice of psychotherapy was mainly a means to an end (i.e., a way to make an income), rather than the primary goal of the sojourn in Sweden. However, after having eventually perhaps secured a permanent residence permit in Sweden, this occupation could be practiced officially and become an important element of the interviewee’s “new life.” A particular goal that was expressed by the interviewee was to have her own psychotherapeutic clinic in Sweden. However, to only work part time was regarded as a viable option as the interviewee had predominantly “traditional” family values which reflect the hybrid home culture which is signified by a blend of tradition and modernity (Edelbay, 2012). In other words, a future husband may preferably be the primary breadwinner of the household. The interviewee explains this as follows:

I expect my boyfriend to take care of me, especially when I don’t have much of an income here. He doesn’t have to be rich but not stingy. So far, I have only met guys who behave like this, who share a more ‘non-Swedish’ mindset, whether they are ethnic Swedes or not. There are such men here but they are probably rare. Moreover, I cannot really accept a feminine man. It does not mean that all Russian or Kazakh men are better or preferable, but that is just how things are in my culture. I cannot change that.

Thus, the interviewee has managed to absorb or “regenerate” the traditional-leaning conception of a heterosexual relationship in a more liberal context, which manifests the interdependence between personality and culture and between the country of origin and the new country of sojourn which intersect. The interviewee continues the discourse. The following quotes underscore the benefits and flaws of Kazakhstan compared to the life and culture of Sweden:

But I don’t mean that I should expect everyone to accommodate to me. Sure, I don’t like everything about Sweden. It is well-organized but also a bit boring. I miss some of the food and other qualities of my home country, such as the quality of service at restaurants and even the nightlife. On the other hand, Sweden has a much better air quality and nature which is clean, safe, and accessible. The traffic and transportation are much better in Sweden, too.

In fact, the drastic improvements of air quality, traffic, and everyday behavior among citizens were some of the reasons why the interviewee really wanted to stay in Sweden permanently. The interviewee’s meaning-making process implies that the material and environmental benefits of Sweden constitute a strong advantage, something that can be compared with the less developed country of origin. It might be a part of a developmental discourse in which, for example, air quality constitutes a positively connotated nodal point. Furthermore:

I want to have a good income and am rather ambitious. I have ideas and ambitions. The first step is to study to validate my earlier certificates, then become an official psychotherapist and then have my own clinic whenever that is possible. It will take time, of course. Well, who knows, I might even have a higher income than my husband in the future.

These quotes do also highlight some of the symbolic boundaries between the old and the new. On the one hand, there are things from the home culture that “cannot be changed” as regards the cultural-psychological constitution of the interviewee but on the other hand, there are unhesitatingly conditions and elements that appear better and perhaps more important than expected in the new location. The interviewee does often mention the pros and cons of Sweden compared to Kazakhstan and Russia. This may appear similar to cultural hybridization and acculturation (i.e., partial acceptance or rejection), but it might imply a form of semiotic and culturally novel perspective which is more congruent with proculturation. When I listened to the interviewee’s discourse, it struck me that she occasionally offers “fresh” and nuanced critique of the Swedish society in regard to some of its flaws and benefits.

In the Swedish context, it is possible to make a transition (“spårbyte”) from asylum seeker to work permit seeker, but the success in that pursuit requires that an individual is connected to a Swedish worker’s union and has worked in the same field (e.g., elderly care) for 4 consecutive years (Swedish Migration Agency, 2022). As the interviewee did not meet the basic criteria for obtaining an asylum-related residence permit, a decision which was established and finalized after three rounds of appeals against this verdict over the course of approximately 5 years, the interviewee tried to make a work transition but could not demonstrate a continuous work situation that would enable such a successful shift. In fact, the interviewee had only worked unofficially as a psychotherapist, typically earning little income, during and after the Covid-19 period (i.e., early 2020 onwards). During the first years in Sweden, she also worked as a waiter at a restaurant, but the employer mistreated her and did not provide any employment certificate that could be useful and important in the application process. Because Sweden requires a permit and basic language-related education (SFI, Svenska för invandrare, i.e., Swedish for migrants) for a migrant psychotherapist to practice the profession officially, this was not an option for the interviewee.

What is interesting in this specific respect is not that the person in question tried to obtain a residence permit in Sweden and was unsuccessful in that pursuit (many migrants are, especially after the slightly more restrictive policies and practices were implemented after 2015) but how the person managed to practice the profession as a psychotherapist despite the above-mentioned judicial and practical obstacles. This theme will be further examined in the next section.

### New Technologies as Adaptive Cultural, Psychological, and Economic Tools

As Gamsakhurdia (2018, 2019) stresses, the culture in sending country A is typically not completely different from its counterpart in the receiving country B. Whereas Sweden unhesitatingly is a more well-developed country as regards, for example, economic and democratic elements, Kazakhstan is nowadays not a poor country per se. People there, especially in larger cities like Almaty and Nur-Sultan, use smart phones virtually every day. For example, in 2015 the Internet usage rate was above 70% (Our world in data, 2022). Those who could afford it wear fashionable western brands like Gucci or Chanel.

Thus, when the interviewee came to Sweden in late 2015, it did not come as a mind-blowing chock as regards the differences in culture, technology, or living standards. Moreover, the interviewee had previously travelled to, for example, Dubai and the USA with her parents, whose socioeconomic status is to be regarded as upper-middle class by Kazakhstan standards and thus experienced some degree of a more cosmopolitan lifestyle.

Nonetheless, the interviewee experienced work conditions vastly different from both the ones that are common in Kazakhstan and in Sweden as regards the psychotherapy profession, in that all sessions were carried through online through the video call function in the popular communication app WhatsApp. The interviewee explains this as follows:

When I have a patient [From for example Russia or Kazakhstan, the author’s comment], the session lasts for 50 minutes. My patients prefer video calls over phone calls. Because of the time difference [typically a 4 or 5 hours difference between Sweden and Kazakhstan/Russia, the author’s comment], I often get up at dawn to talk to the patients. But it varies a lot. Apart from that, it is like a regular therapy session.

As a rather unexperienced psychotherapist, currently in her late 20 s, she had yet to decide the specific direction of profession as regards psychotherapeutic perspectives (e.g., CBT, psychodynamic, Gestalt). The interviewee did also convey that payment transactions can be a bit difficult to carry through, both in general and in relation to the profession. In Sweden, migrants such as asylum seekers share most of the civil rights and liberties with Swedish citizens, but it is not possible to possess a BankID application, which is commonly used for transactions and payments these days in Sweden.

When asked about the possibility to treat and interact with patients with a Swedish background, the interviewee underlined that the language ability in Swedish is yet too poor at this point for her to be able to talk about complex issues often embedded in the psychotherapeutic field (“My Swedish is simply not good enough yet”). She also conveyed that the English abilities were inadequate. Therefore, only Russian is used during the psychotherapy sessions as no Swedish clients have been recruited. Currently, the client base does exclusively consist of patients who reside in former Soviet states (e.g., Russia, Kazakhstan), although she had had a few sessions in Sweden with Russian-speaking clients. As said, a future Swedish citizenship, or at least a permanent residence permit, is required for the interviewee to practice the profession officially and with a possibly broader client base in Sweden. The validation of the psychotherapy certificate from Kazakhstan requires that the interviewee is already qualified as a permanent residence permit holder who can participate in the introductory SFI language training program and additional tertiary studies. This leaves migrants under these and similar circumstances in a catch 22 situation.

The economic and national differences between a wealthy nation like Sweden on the one hand and emerging countries like Kazakhstan and Russia on the other hand do also have direct implications in relation to the interviewee’s transnational profession and socioeconomic situation. For instance, the annual per capita average income in Kazakhstan is 8820 US dollars, 11,220 in Russia, and 55,820 in Sweden (World Data, 2021). This means that the interviewee, whose clients are located in comparatively poorer countries, can only work for low hourly wages but still live in a country (Sweden) where the price levels are based on a much higher average income level. Fortunately, the interviewee has received help from a relative who lives in Sweden and previous boyfriends, at whose apartments the interviewee had lived for extensive periods during the sojourn in Sweden. Moreover, the recruitment of Russian speakers in Sweden enables a standard rate similar to those of Swedish psychotherapists but that only happened at a late stage of the sojourn.

At this point, the transnational psychotherapy practice indicates that psychological, cultural, and economic flows go from B (Sweden) to either A (Kazakhstan) or C (Russia) and then the money paid for such sessions go back to A. But some of these processes are also bidirectional, as the patients’ discursive elements stem from A or C and flow to B. As Vygotsky (e.g., 1971, 1987) noticed, various cultural tools are important for psychological growth and development. Similarly, technological tools such as WhatsApp have been pivotal for the unofficial but nevertheless real practice of the psychotherapy profession for the interviewee in the post-migration Swedish context. Such cultural, psychological, occupational, and economic practices have a transitional character in that sense that the main cultural and technological tool (WhatsApp) is transnational per se (it has no specific national boundaries, as long as it is not blocked in a particular location), that the discursive and semiotic flows are transnational, and that the main subject (the interviewee) has moved from Kazakhstan to Sweden but still works with clients in Kazakhstan, Russia, and a few other former Soviet states. These conditions were also present pre-Covid-19 for the interviewee, whereas during the pandemic, online counseling has become pervasive for psychotherapists in general (e.g., Swartz, 2020).

### Cultural and Individual Identity

With regard to the cultural and religious identity of the interviewee, the following quote accentuates the specific subjective position:

I am a Muslim but I also like to go to the church. When I am in a mosque in Kazakhstan, I don’t feel so much. I have never visited a mosque in Sweden but a few churches. But still Islam is important for me and especially my father. He is a better man these days as a Muslim, he works hard and never drinks alcohol. He treats my mother with great respect. I never eat pork, partly to honor him.

**Fig. 1:**
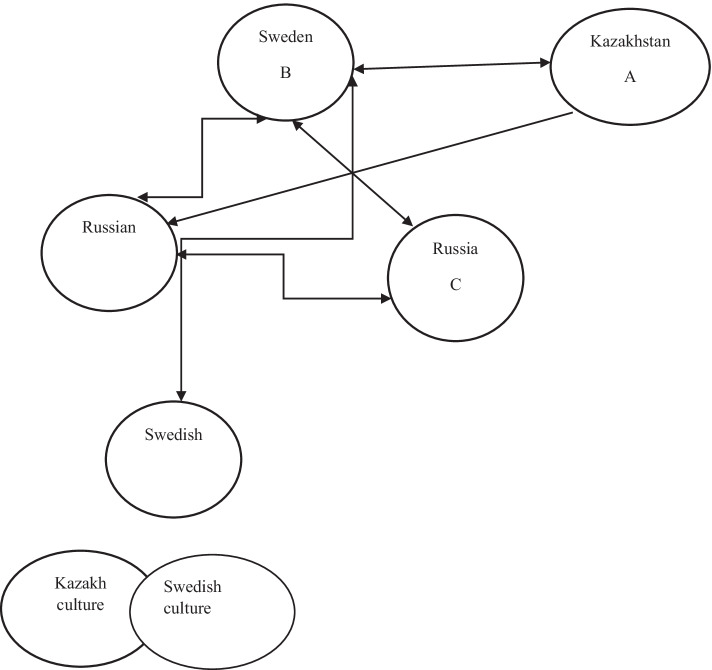
Semiotic flows and cultural identity markers. Notes: One-headed arrows signify a one-directional relationship, such as from A to B, whereas two-headed arrows signify bidirectional or reciprocal relationships, such as semiotic occupational flows. The lower section of the figure illustrates the procultural, partial overlap, or mix between Kazakh and Swedish culture for the interviewee

This signifies a form of proculturation dynamics that is far from unilinear in that sense that the main subject (X) has migrated from A to B and then gradually become “proculturated” and absorbed some elements of the new Swedish host culture. Such dynamics, complexities, and nuances are expressed in three major ways:

1. First and foremost, the interviewee does still identify as a female Kazakh and a-religious Muslim (but not completely a-religious), whose first language and current work language is Russian (Kazakh is seldomly used). These basic elements of the cultural identity remain intact.
2. Secondly, Swedish culture has been absorbed but only partially. For example, the interviewee’s alcohol consumption increased during the first years in Sweden which reflects the more secular and liberal culture of Sweden compared to Kazakhstan (e.g.,, Boman, 2021b; Edelbay, 2012). Moreover, the interviewee has managed to learn Swedish to a moderate level within the span of approximately 6 years but without the aid from official education programs such as SFI or tertiary institutions. It is likely that the frequent encounters with the Swedish Migration Agency have affected the learning processes but also personal relationships.
3. Thirdly, the occupational situation is characterized by various transnational and transcultural flows, whose semiotic processes include subjects located in different countries, as well as crisscrossing of cultural, psychological, and economic flows (e.g., see Castells, 2004; Gamsakhurdia, 2018, 2019). This is illustrated in Fig. 1.

Hence, the proculturation concept catches these semiotic dynamics and lived experiences in a more meaningful way than acculturation does, which is mostly concerned about gradual acceptance or rejection of a new national culture of migrant(s) X (Gamsakhurdia, 2018). The production of cultural novelties implies the new perspectives of the Swedish society’s benefits and shortcomings but also the contribution to the psychotherapy profession. Although unofficially, a Russian-speaking psychotherapist have much to offer Russian speakers in Sweden but also the experiences from the home country. As Valsiner (2014) emphasizes  in regard to qualitative cultural psychology, compared to mainstream quantitative cross-cultural psychology, there is no complete separation between the dependent and the independent variables as these are regarded as *interdependent* factors. Although the impact of a particular migrant on a host society is typically very limited, whereas the opposite direction implies a large impact (i.e., the host culture does typically affect the individual migrant quite substantially), the total impact of all migrants on the host culture might be considerable. Hence, in relation to the procultural trajectory of a single migrant, it is rather the new host society which influences the migrant rather than the other way around. However, the individual migrant’s meaning-making processes in the intersection and interaction between the old and new may contribute with novel understandings and occupational competence in the new location.

One may also reiterate that in the Swedish context, the migration and integration policies are rather lenient and liberal (Beck et al., 2017; Sanandaji, 2020). Thus, migrants are typically not imposed the Swedish host culture to the same extent as the counterparts in other countries. Also, some identity markers such as ethnic background, primary language, and citizenship are fairly fixed elements and not subject to any drastic change after migration to a different country and culture has occurred, especially at an adult age (e.g., Kwak, 2010). Be that as it may, it is fairly easy to maintain substantial elements of the country/culture of origin such as parlance, ethnocultural friend groups and social networks, religious beliefs, as well as most if not all clothing preferences. But from a mere functional perspective, the interviewee’s flexibility between the old and new cultural ingredients was particularly useful. Instead of leaving the Russian language behind, it was absolutely necessary to use it in relation to the transnational psychotherapy professional practice. Basic to moderate skills in Swedish were of some use in the current vulnerable and essentially non-existent position in the Swedish labor market, while the interviewee worked as a restaurant waiter. In many occupational contexts, abilities in Swedish would have been beneficial but as regards the primary occupation as a psychotherapist Swedish was not useful. Instead, Swedish was helpful in a variety of everyday life contexts such as interacting with Swedish friends, staff members at various locations, and the Swedish Migration Agency.

Because of the Covid-19 pandemic, the interviewee was granted some additional time in Sweden before the compulsory return process was implemented and she had to leave the country. The plan is still to form a life in the “new” country, and due to the recent outbreak of violent protests in January 2022 and the Russian invasion of Ukraine February 24 onwards, a future in Kazakhstan (a partner country to Russia) does perhaps seem more unpleasant than ever. This might also facilitate the practice of the profession as a psychotherapist under more economically feasible and favorable conditions.

## Conclusion and Discussion

The current article has looked into the specific life situation of a relatively young female Kazakh migrant in Sweden, as regards the person’s life experiences, cultural identity, and specific and rather precarious work conditions as a psychotherapist in Sweden. The interviewee had obtained a psychotherapy degree and certification from Kazakhstan, but as an asylum seeker, it was not possible to practice the profession officially. Instead, this individual used the communication app WhatsApp to carry through psychotherapeutic counseling sessions with patients in, for example, Kazakhstan and Russia. This echoes Vygotsky's (e.g., 1971, 1987) ideas about cultural tools. In this case, the communication app functioned as a technological, cultural, economic, and psychological and not the least a communicative tool that solidified the interviewee’s professional identity as a psychotherapist and practitioner and as an economic instrument to earn income. Perhaps, the psychotherapeutic field, in Sweden and elsewhere, may consider expanding the client base to other countries and languages, although in official contexts. Basic mobile and computer applications such as WhatsApp and Zoom are useful for the communication with clients in that regard, whereas transnational monetary transactions could be carried through by means of, for example, PayPal. Fortunately, this is easier when regular practitioners, compared to asylum seekers, have access to all rights and privileges as citizens.

Whereas the cultural identity was solid in regard to basic identity markers (e.g., woman, Kazakh, Muslim) and malleable and flexible in terms on language use (Russian, Swedish), the work conditions were signified by a transnational crisscrossing of semiotic and discursive flows between Sweden, Kazakhstan, Russia, and a few other post-Soviet states. This was because the person’s ability in Swedish was not sufficient to interact with predominantly Swedish-speaking patients/clients, and therefore the latter were Russian-speaking individuals. However, when the person worked as, for example, a restaurant staff, basic Swedish was used.

Partly similar to Kwak (2010) experiences as a migration scholar from South Korea who moved to Canada (Toronto), the interviewee in the current article experienced a more liberal society in Sweden (Stockholm) compared to Kazakhstan (Almaty) which was signified by increased alcohol consumption during the years of residence in Sweden (which lasted between 2015–2022). On the other hand, as Gamsakhurdia (2019) underlines, these cultural differences are relative rather than immensely striking. For example, the a-religious Muslim identity which is typical for Kazakh people in general (Edelbay, 2012; Spehr & Kassenova, 2012) had already laid the foundation for (increased) alcohol consumption in a more liberal cultural and national context.

Several migration studies relevant for the Swedish context (e.g., Manhica et al., 2018; Vogiazides & Mondani, 2019) demonstrate that migrants experience difficulties in regard to finding their first jobs. However, this picture is fuzzy because the interviewee could not work officially as a psychotherapist in Sweden but could work officially with other types of jobs of lower status (e.g., restaurant staff, elderly care worker, nanny) in the Stockholm region, which also could be a potential path to a residence permit after several years of consecutive work in the same field. The interviewee was well aware of the different paths and possibilities and did often praise her two lawyers at the Swedish Migration Agency, one in particular, for their efforts to help her. Female migrants might be vulnerable in many ways (e.g., Manhica et al., 2018) and the interviewee described some difficulties (e.g., a few other migrants had tried to use her as an extremely low-paid but full-time working nanny in exchange for a small room), but there were many examples of the opposite. Overall, she considered the Swedish majority group positively, although with some exceptions.

Moreover, the interviewee could appeal against the asylum rejection three times and was given additional time after the definitive decision was announced in the first half of 2021. Even after that decision she could find ways to stay in Sweden even longer, thus postponing the return to Kazakhstan.

Nevertheless, this person is currently in a rather precarious situation. Thus, the future trajectory of the interviewee remains uncertain. Nonetheless, it is evident that the person has kept some “essential” cultural elements from the home country (Kazakhstan) and added some from the receiving country (Sweden), which indicates a procultural constitution of the identity that transcends mere acceptance or rejection of the culture in the new location (Gamsakhurdia, 2018). Further research of a similar direction may include more individuals from the same country or compare individuals from different countries and cultures.

## Data Availability

No specific data set that could be shared publicly, and as was stated in the manuscript text that is due to ethical concerns, is associated with this manuscript.
